# Modification of the second translation initiation site restricts the replication of foot-and-mouth disease virus in PK-15 cells

**DOI:** 10.1007/s00253-020-10810-w

**Published:** 2020-08-21

**Authors:** Hong Yuan, Na Li, Pinghua Li, Xingwen Bai, Pu Sun, Huifang Bao, Xiaohua Gong, Xueqing Ma, Yimei Cao, Kun Li, Yuanfang Fu, Jing Zhang, Dong Li, Yingli Chen, Jie Zhang, Zengjun Lu, Zaixin Liu

**Affiliations:** State Key Laboratory of Veterinary Etiological Biology, OIE/China Foot-and-Mouth Disease Reference Laboratory, Lanzhou Veterinary Research Institute, Chinese Academy of Agricultural Sciences, No. 1 Xujiaping, Yanchangbao, Lanzhou, Gansu 730046 People’s Republic of China

**Keywords:** FMDV, Vaccine, Virus replication, Translation initiation, Leader protein

## Abstract

**Abstract:**

The translation initiation of foot-and-mouth disease virus (FMDV) occurs at two alternative initiation sites (Lab AUG and Lb AUG). Usually, the Lb AUG is more favorably used to initiate protein synthesis than the Lab AUG. To explore the effect of Lb AUG on FMDV replication and obtain FMDV with restricted replication, this initiation codon was mutated to a variety of non-AUG codons (UGG, AUC, CUG, and AAA). Fortunately, the modifications did not prevent viral viability but influenced replication characteristics of some FMDV mutants in a cell-specific manner, as was shown by the similar replication in BHK-21 cells and delayed growth kinetics in PK-15 cells. This attenuated phenotype of FMDV mutants in PK-15 cells was found to be correlated with reduced abilities to cleave eIF4GI and suppress interference (IFN) expression. As leader (L) protein was reported to be responsible for eIF4GI cleavage and inhibition of IFN expression, the in vivo L protein synthesis was examined during the infection of FMDV mutants. Our results showed that not only the total yield of L proteins was severely influenced but also the individual yield of L protein was seen to be affected, which implied that both the relative usage of the two initiation sites and overall translation efficiency were changed by Lb AUG modifications. In addition, the in vitro translation activity was also negatively regulated by Lb AUG mutations. Collectively, these findings suggested that the restricted replications of Lb AUG-modified FMDVs were related to the delayed eIF4GI cleavage and decreased ability to block IFN expression but were mainly determined by the inefficient translation initiation. FMDVs precisely with modifications of Lb AUG initiation codon may represent safer seed viruses for vaccine production.

**Key points:**

*• The polyprotein translation of FMDV initiates at two alternative initiation sites (Lab AUG and Lb AUG). In order to explore the effect of Lb AUG on FMDV replication and obtain FMDV with restricted replication, the Lb initiation AUG was mutated to a variety of non-AUG codons (UGG, AUC, CUG, and AAA), and four FMDV mutants with Lb AUG modification were generated.*

*• We found that partial FMDV mutants grew almost as well as WT virus in BHK-21 cells, a typical cell line used for FMD vaccine production, but displayed impaired replication in IFN-competent PK-15 cells.*

*• The attenuation of mutant FMDVs in PK-15 cells was found to be correlated with delayed eIF4GI cleavage and decreased ability to block IFN expression.*

*• We proved that the attenuated phenotype of Lb AUG-modified FMDVs was mainly determined by the inefficient translation initiation, as demonstrated by the decrease of total yield of L proteins and individual production of L protein.*

*• We successfully generated genetically engineered FMDV with attenuated phenotype. The approach of precise engineering of FMDV with the modification of initiation codon provides a safe platform to produce inactivated antigen vaccines.*

## Introduction

Foot-and-mouth disease (FMD), caused by foot-and-mouth disease virus (FMDV), is a highly infectious disease of domestic and wild cloven-hoofed animals including cattle, swine, sheep, and goats (Grubman and Baxt 2004). Outbreaks of FMD can have severe economic and social consequences in affected countries as demonstrated by the 1997 FMD outbreak in Taiwan and the 2001 outbreak in the UK, making FMD the most economically important disease of livestock worldwide (Scudamore and Harris 2002; Thompson et al. 2002; Yang et al. 1999). Currently, the most effective control policy of this disease is mainly achieved by vaccination with inactivated whole virus as well as slaughter of infected and exposed animals. However, the production of inactivated vaccine requires expensive high-containment facilities where live FMDVs are grown in large volumes, which poses a risk of virus escape from the manufacturing facilities (Cottam et al. 2008; King et al. 1981). Indeed, there does have been an association of disease outbreaks with incomplete chemical inactivation and escape of virus from vaccine manufacturing facilities (Cottam et al. 2008). To overcome this serious production risk, one of the approaches is to develop attenuated FMDV strains. Previous works suggested that the impaired growth of FMDV was more responsible for the virus attenuation (Arzt et al. 2017). Therefore, it is crucial to obtain FMDV with reduced replication.

FMDV contains a single-stranded, positive-sense RNA genome with the length of about 8500 nucleotides (Belsham 2005). Leader (L) protein, a papain-like protease, is a virulence factor encoded by FMDV. This protein, located at the N terminus of the polyprotein, is produced in two forms: termed Lab and Lb. The two proteins differ in their N termini since the initiation of viral protein synthesis occurs at two distinct sites, separated by 84 nucleotides (La region) (Clarke et al. 1985; Sangar et al. 1980). Both forms of L proteins exhibit similar activities with respect to the cleavage of L/P1 junction and efficient degradation of eukaryotic translation initiation factor 4G (eIF4G) which is responsible for the translation of capped mRNAs in host cells (Devaney et al. 1988; Gradi et al. 2004; Medina et al. 1993; Strebel and Beck 1986). It also has been reported that Lb protein correlates with the pathogenesis in the natural host and is involved in suppressing host innate immune responses via multiple strategies (Chinsangaram et al. 1999; de Los Santos et al. 2006, 2007; Wang et al. 2011a, b; Zhu et al. 2010). Since L protein plays a crucial role in FMDV pathogenesis, manipulation of the L coding sequence might allow us to derive a viable attenuated mutant FMDV.

The two AUG initiation codons are relatively conserved in seven serotypes of FMDV, and the selection of the two initiation sites varies between FMDV strains (Belsham 1992; Clarke et al. 1985; Sangar et al. 1987). A widespread model of the selection mechanism of translation initiation sites in FMDV mRNA, which is similar to that in encephalomyocarditis virus (EMCV), suggests that the 40S ribosome is initially recruited to the Lab site, and the Lb site is assessed by linear scanning (Andreev et al. 2007; Lopez de Quinto and Martinez-Salas 1999; Poyry and Jackson 2011; Zhou et al. 2010). Another observation suggests that some ribosomes access the functional initiation site by a nonlinear shunting-like mechanism (Poyry et al. 2001). A number of factors have been found to determine the selection of each initiation codon: (I) the surrounding nucleotide sequences (Kozak 1997; Ma et al. 2016; Nakagawa et al. 2008; Pisarev et al. 2006; Zhou et al. 2010). According to the nucleotide sequences flanking the two initiating AUGs, FMDV strains have been categorized into three groups: One group, represented by virus A10, has a favorable sequence context at both Lab and Lb sites; the second group, consisting of the majority of FMDVs, possesses an inefficient initiation at Lab site as well as an efficient initiation at Lb site, while accompanied by little or no Lab product in vitro; the third group, as shown by virus 01 BFS and 01 Kaufbeuren, has an unfavorable Lab AUG and a favorable Lb AUG, whereby producing detected Lab and Lb products in vitro (Sangar et al. 1987): (II) the Mg2^+^ concentration in conditions. The slightly elevated Mg2^+^ concentrations increase in vitro initiation at AUG codons which have a poor local sequence context (Sangar et al. 1987): (III) the RNA secondary structure of La region (Andreev et al. 2007; Lopez de Quinto and Martinez-Salas 1999; Poyry and Jackson 2011). The elimination of RNA secondary structure downstream of the Lab site is found to dramatically reduce the initiation at the Lab site with a compensatory increase in initiation at the Lb site (Poyry and Jackson 2011). Stabilization of a stem-loop in front of Lab AUG also affects the efficiency of start recognition (Andreev et al. 2007): (IV) the codon in both the Lab and Lb sites (Andreev et al. 2007; Lopez de Quinto and Martinez-Salas 1999; Poyry and Jackson 2011). For example, mutating the Lab site to RCG or RUN codons stimulates Lb initiation by 20–40% (Poyry and Jackson 2011).

As the selection of initiation codon is the core of translation initiation which influences translation efficiency, the two initiation sites in the L coding sequence must play vital roles for FMDV replication. A previous study has analyzed the effect of mutation of each of the two initiation AUGs on viral replication, and the results indicate that a mutation of Lab AUG to UGG does not prevent the production of viable FMDV, but the modification of Lb AUG to UUU abolishes virus replication in BHK-21 cells (Cao et al. 1995). Another investigation has shown that an in-frame 57-nt transposon insertion in La region results in a decreased translation initiation from the Lb AUG, causing reduced virulence in cattle (Piccone et al. 2010). Furthermore, insertion of an epitope tag (Flag) or a small tetracysteine motif into La region does not influence virus survival but abolishes or greatly diminishes initiation at the Lab AUG in these viruses (Piccone et al. 2011). These earlier results demonstrate that the relative usage of Lab and Lb initiation sites is crucial for FMDV proliferation.

To date, there is no available FMDV with Lb AUG modification. In order to explore the influence of Lb AUG on FMDV replication and obtain attenuated FMDV, we generated and characterized four FMDV mutants in which the Lb AUG was modified, respectively. We also explored the detailed mechanism for the impaired replication of Lb AUG-modified FMDVs in PK-15 cells. Our current works confirmed that Lb AUG modifications resulted in attenuated phenotypes of genetically engineered FMDV mutants in IFN-competent cells fundamentally through impairing the translation initiation efficiency of viral polyprotein. The approach of precise engineering of FMDV with the modification of Lb initiation codon not only contributes to addressing the individual role of Lab protein in FMDV replication and pathogenesis but also offers the possibility to develop safer vaccines.

## Materials and methods

### Cells, viruses, plasmids, and antibodies

Baby hamster kidney (BHK-21) cells and pig kidney (PK-15) cells were kept in the laboratory of the Lanzhou Veterinary Research Institute (LVRI; Lanzhou, China). BHK-21 cells were used to prepare virus stocks and calculate virus titer. BSR/T7 cells (Rieder et al. 1993) were utilized to recover the modified viruses as described previously (Li et al. 2014). FMDV O/HN/CHN/93 was derived from the infectious cDNA clone pOFS (Li et al. 2012b; Yuan et al. 2020). The psiCHECK™-2 vector was purchased from Promega (Madison, WI, USA). Anti-eIF4GI rabbit polyclonal antibody and anti-Flag mouse monoclonal antibody were purchased from Sigma-Aldrich (St. Louis, MO, USA); anti-β-actin mouse monoclonal antibody was purchased from Santa Cruz Biotechnology Inc. (Dallas, TX, USA); anti-FMDV pig polyclonal serum and mouse monoclonal antibody MAb 3A24 against FMDV 3A were provided by OIE Reference Laboratory of China (Lanzhou, China); rabbit polyclonal serum against L fusion protein, which could especially identify FMDV-encoded L protein in BHK-21 cells through immunofluorescence and Western blot analyses, was prepared by our lab (data were not published); the horseradish peroxidase (HRP)-conjugated secondary antibodies were purchased from Cell Signal Technology (Danvers, MA, USA).

### Construction of plasmids

In order to analyze the function of Lb AUG, four plasmids with different modifications of Lb AUG (TGG, ATC, CTG, and AAA) were constructed based on the previously mentioned plasmid pSK-Z123 by site-directed mutagenesis (Li et al. 2012b). Table 1 listed the primers used for Lb AUG mutation. The modified plasmids, which were confirmed by sequencing, were digested with *Spe* I and *Bgl* II and constructed back between *Spe* I and *Bgl* II sites of the full-length cDNA pOFS for FMDV O/HN/CHN/93, producing recombinant full-length plasmids pLab3m, pLab4m, pLab5m, and pLab9m. The resultant plasmids were verified by sequencing.

**Table 1:** Primers used for the mutagenesis of Lb AUG initiation codon

Name	Sequence (5′–3′)
*TGG*-F	TCACGAACACAAGGGAAAtggGAATTCACACTTCACAAC
*TGG*-R	GTTGTGAAGTGTGAATTCccaTTTCCCTTGTGTTCGTGA
*ATC*-F	TCACGAACACAAGGGAAAatcGAATTCACACTTCACAAC
*ATC*-R	GTTGTGAAGTGTGAATTCgatTTTCCCTTGTGTTCGTGA
*CTG*-F	TCACGAACACAAGGGAAActgGAATTCACACTTCACAAC
*CTG*-R	GTTGTGAAGTGTGAATTCcagTTTCCCTTGTGTTCGTGA
*AAA*-F	TCACGAACACAAGGGAAAaaaGAATTCACACTTCACAAC
*AAA*-R	GTTGTGAAGTGTGAATTCtttTTTCCCTTGTGTTCGTGA

Both the internal ribosome entry site (IRES) and mutant L fragments, which were obtained from PCR products, were introduced at *Pme* I and *Apa* I sites of psiCHECK™-2 vector to produce psicIRESWT, psicIRESLab3m, psicIRESLab4m, psicIRESLab5m, and psicIRESLab9m, respectively. The detailed construction processes were described previously (Han et al. 2020; Huang et al. 2012). The introduced fragments were analyzed by sequencing as well.

### Rescue of mutant viruses

The four recombinant plasmids in addition to the control plasmid pOFS (2 μg), which were linearized by *Not* I, were transfected into BSR/T7 cells using Lipofectamine^TM^ 2000 (Invitrogen). The transfected cells were incubated at 37 °C for 48 h, and the supernatants were harvested following freezing. The viruses recovered from transfections were amplified by 6 rounds of infection of BHK-21 cells. Virus titers were determined in BHK cells by calculating the 50% tissue culture infectious dose (TCID_50_) as described previously (Li et al. 2012b). The viral RNA was extracted from the passage 3 and 6 of each recovered virus using RNeasy mini kit (Qiagen) according to the manufacturer’s manual, and the remaining DNA was eliminated by digestion with RNase-free DNase I (Qiagen). One-step reverse transcription-polymerase chain reaction (RT-PCR) was employed to amplify a cDNA fragment including L region by synthetic oligonucleotides as follows: (LF) 5′-ACAAACACGGTCTAAGCAGG-3′; (LR) 5′-GTTCTGGTATTGCTGCAT-3′. The resulted fragments were sequenced using the following primer: (LCF) 5′-AGGTAACACGAGACACTC-3′. These results were confirmed by at least two independent sequencing reactions.

### Immunofluorescence assay

BHK-21 cell monolayers were grown on 20-mm glass-bottomed culture dish (NEST) and infected with modified FMDVs at a multiplicity of infection (MOI) of 10 for 4 h. Cells were fixed in 4% paraformaldehyde, permeabilized with 0.5% Triton X-100 (Sigma-Aldrich, St. Louis, MO, USA) in phosphate-buffered saline (PBS), and then blocked with 1% bovine serum albumin (BSA) overnight at 4 °C. FMDV 3A protein was detected with primary antibody MAb 3A24 and Cy3-conjugated secondary antibody, and nuclei were detected with 4′,6-diamidino-2-phenylindole (DAPI) staining (Beyotime Institute of Biotechnology, Shanghai, China). Eventually, Alexa Fluor 405 and Alexa Fluor 561 were examined using a laser-scanning confocal microscope (LSCM; Leica SP8, Leica Camera AG, Wetzlar, Germany).

### The growth of FMDV mutants in culture cells

To analyze the effects of Lb AUG mutations on FMDV replication, plaque morphology of modified FMDVs was evaluated in BHK-21 or PK-15 cells as described previously (Bai et al. 2019; Li et al. 2012a; Vazquez-Calvo et al. 2014). The virus-infected cells were stained at 48 h post-infection (hpi). Then, the growth curves of modified FMDVs were assayed. BHK-21 or PK-15 cells were infected with viruses at 1 MOI, and the samples were harvested at 3, 6, 9, and 20 hpi. After one freeze/thaw cycle, virus titers were determined by TCID_50_. All experiments were performed in triplicate.

### Western blot analysis

PK-15 cells infected with modified FMDVs at 1 MOI were harvested for 6 h. The collected samples were lysed and separated by 4–20% sodium dodecyl sulfate-polyacrylamide gel electrophoresis (SDS-PAGE). Resolved proteins were transferred onto polyvinylidene difluoride (PVDF) membranes (Millipore, Burlington, MA, USA) and interacted with anti-eIF4GI rabbit polyclonal antibody (dilution, 1:250), anti-FMDV pig polyclonal sera (dilution, 1:1000), and anti-β-actin mouse monoclonal antibody (dilution, 1:1000), followed by HRP-conjugated secondary antibodies. The proteins immobilized on a membrane were detected by Western Lightning® Plus ECL (PerkinElmer, Waltham, MA, USA).

For the detection of synthesized viral proteins, PK-15 cells were infected with FMDV (MOI = 1) and harvested at different time points (1, 4, 8, 12, and 20 hpi). Samples were analyzed by Western blotting as described above. The synthesized L proteins or structural proteins of FMDV were detected by anti-L rabbit polyclonal serum or anti-FMDV pig polyclonal serum. All experiments were performed in duplicate.

### Quantitative RT-PCR (RT-qPCR)

PK-15 cells infected with FMDVs (WT, K3m, K4m, K5m, and K9m) for indicated times were harvested and lysed in dishes. Total RNA was extracted using the RNeasy mini kit. Cellular DNA was removed by treatment with DNase I. The isolated RNA was reversely transcribed to cDNA by Reverse Transcriptase M-MLV (Takara, Dalian, China). Synthesized cDNA was subjected to quantitative PCR using SYBR green PCR master mix (Takara, Dalian, China) with the ABI 7500 real-time PCR system. The primers used for RT-qPCR were shown as follows: IFN-α forward primer 5′-TGGTGCATGAGATGCTCCA-3′ and reverse primer 5′-GCCGAGCCCTCTGTGCT-3′; IFN-β forward primer 5′-AGTGCATCCTCCAAATCGCT-3′ and reverse primer 5′-GCTCATGGAAAGAGCTGTGGT-3′; IFN-λ1 forward primer 5′-GGTGCTGGCGACTGTGATG-3′ and reverse primer 5′-GATTGGAACTGGCCCATGTG-3′. The GAPDH gene was used as an internal control: GAPDH forward primer 5′-CAAGAAGGTGGTGAAGCA-3′ and reverse primer 5′-AAGTGGAAGAGTGAGTGTC-3′. The relative transcription levels of IFN genes were presented as fold changes relative to the control. All experiments were performed in triplicate.

### Dual-luciferase reporter assay

The constructed reporter plasmids were transfected into BHK-21 or PK-15 cells grown in a 24-well plate, and the samples were harvested at 12 and 24 h post-transfection. Dual-luciferase activities were sequentially measured by Dual-Glo Luciferase Assay System (Promega, Madison, WI, USA). This assay was performed in triplicate.

### Statistical analysis

Data analyses were conducted by Prism (version 5.0) software (GraphPad Software, San Diego, CA) or the Microsoft Excel program. Statistical significances were determined using the unpaired student *t* test, and asterisks indicated statistical significance relative to the nontreatment group (*P < 0.05; **P < 0.01; ***P < 0.001).

## Results

### Generation of FMDV mutants containing modified Lb AUG

**Fig. 1:**
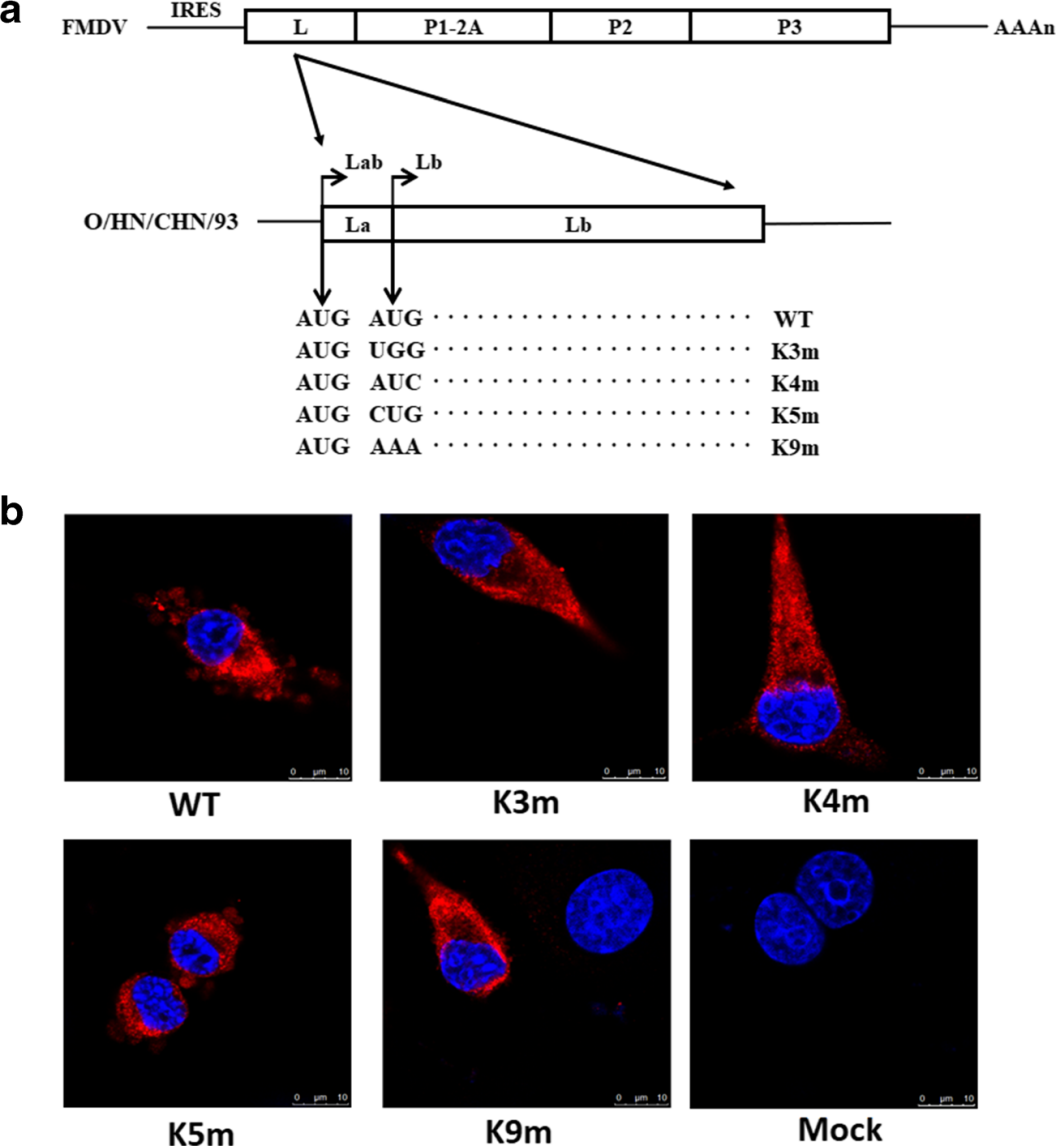
Structure of the modified FMDV genomes (**a**) and immunofluorescence analysis of rescued FMDVs (**b**). **a** The modifications of Lb AUG were individually introduced into the infectious cDNA clone pOFS by site-directed mutagenesis. The wild-type and mutated codons in the two initiation sites (Lab and Lb) were shown. **b** BHK-21 cells were infected with rescued viruses at 10 MOI for 4 h. FMDV protein 3A was detected using mouse MAb 3A24 and Alexa Fluor 561-conjugated secondary antibody

In order to explore the role of Lb AUG, we introduced four mutations of this initiation codon in previously constructed plasmid pOFS. The newly constructed infectious clone plasmids and WT plasmid were transfected into BSR/T7 cells, and five viruses, namely WT, K3m, K4m, K5m, and K9m, were recovered from transfected cells. The genome sequencing of the L coding region in rescued viruses revealed that the resulting viruses maintained the introduced mutations, and no other modification was found. As shown in Fig. 1, BHK-21 cells infected with FMDV mutants could react with the MAb 3A24 antibody, suggesting that FMDV 3A protein was detected during the proliferation of rescued viruses. These findings indicated that four FMDV mutants containing modified Lb AUG were generated.

### Replication of FMDV mutants in cell cultures

**Fig. 2:**
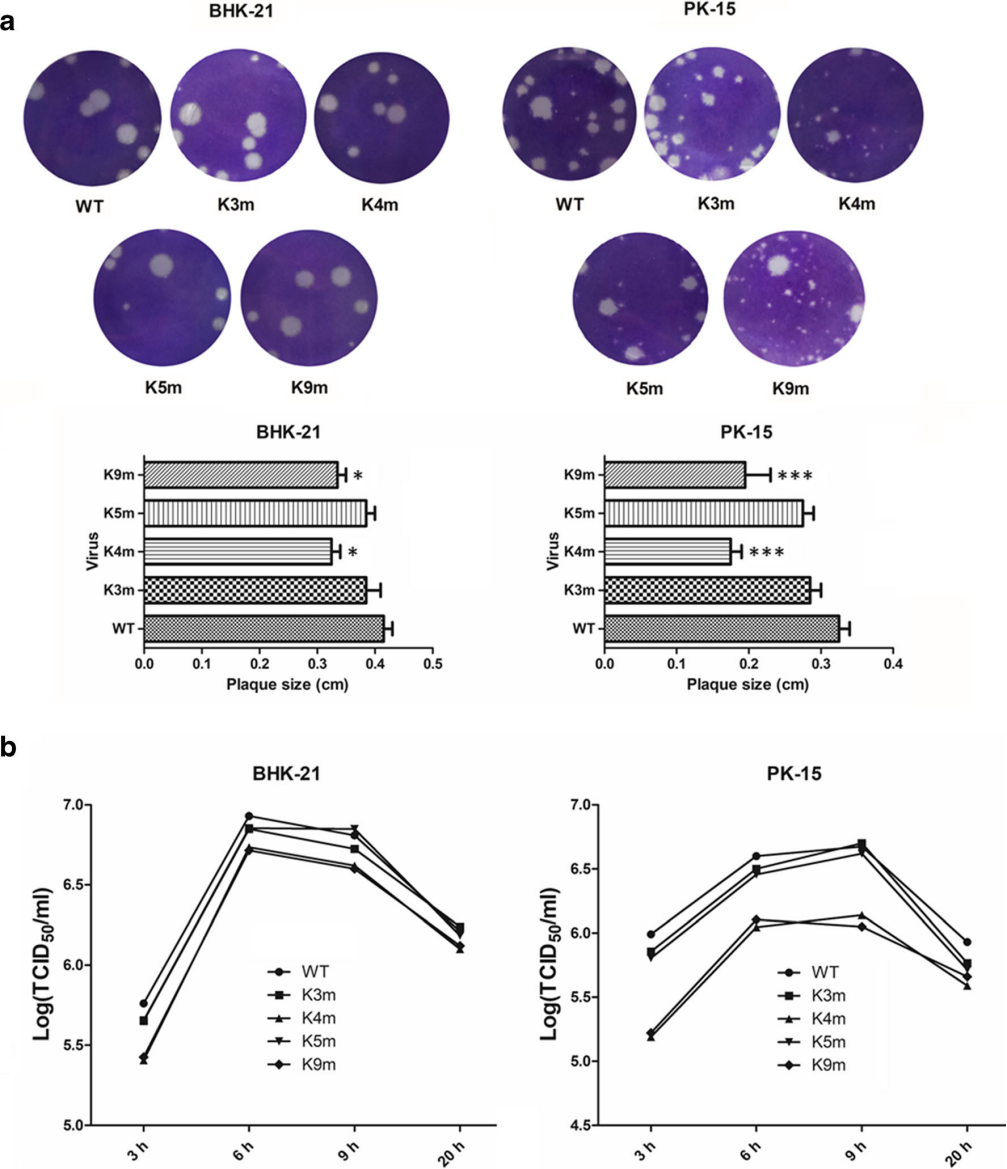
Plaque morphology (**a**) and growth curves of the modified FMDVs (**b**). **a** BHK-21 or PK-15 cells were infected with WT and mutant FMDVs. The cells were stained with crystal violet and visualized at 48 hpi. In order to measure viral plaque size, about 100 plaques were analyzed for each virus. Asterisks (*) denoted statistically significant differences (ANOVA results were indicated [*P < 0.05; **P < 0.01; ***P < 0.001]). **b** The parental and mutant viruses were used to infect BHK-21 or PK-15 cells at 1 MOI. At 3, 6, 9, and 20 hpi, samples were harvested, and virus titer was measured by TCID_50_/ml in BHK-21 cells. Results were expressed as the average values of three independent experiments.

The ability of FMDV mutants to form plaque was assessed in BHK-21 or PK-15 cells as previously described (Fig. 2a). In BHK-21 cells, the four FMDV mutants showed no enormous differences in plaque phenotypes and sizes compared with the WT virus. In PK-15 cells, K3m and K5m had slightly smaller plaque sizes compared with the parental virus (0.1 < *P*), but K4m and K9m formed significantly smaller plaques than the WT virus (*P* < 0.001). The growth kinetics of the modified viruses were also determined in two cell lines (Fig. 2b). All mutant viruses showed no remarkable differences in titers in BHK-21 cells in comparison with the WT virus. Consistent with the plaque phenotype results, in PK-15 cells, K4m and K9m viruses had clearly lower yields than the WT virus (0.001 < *P* < 0.01). No notable delay in the growth of K3m and K5m was visible in PK-15 cells (0.1 < *P*). Taken together, these results suggested that the replication phenotype of FMDV was dependent on the second initiation AUG in L coding sequence.

### Effect of Lb AUG mutations on eIF4GI cleavage in vivo

**Fig. 3:**
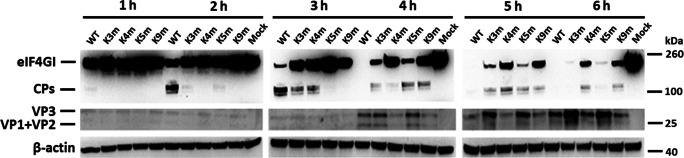
The cleavage of eIF4GI induced by the WT and mutant FMDVs. PK-15 cells were infected with the parental and mutant FMDVs (MOI = 1) and harvested at indicated times (1, 2, 3, 4, 5, and 6 hpi). The eIF4GI cleavage and viral structural proteins were detected by Western blotting. β-actin was measured as a control

In order to investigate the reason for the restricted replication, the cleavage level of eIF4GI was detected within WT- and mutant-infected PK-15 cells. As illustrated in Fig. 3, the eIF4GI was cleaved starting at early stage of FMDV infection, which was consistent with the previous study (Gradi et al. 2004). When comparing with WT virus, the four modified FMDVs exhibited a different level of reduced ability to shut off host cell protein synthesis. We also observed that in accordance with the replication phenotype, cleavage activity within K3m- or K5m-infected cells was more efficiently induced than that within K4m- or K9m-infected cells. These results demonstrated that the Lb AUG was not essential for the cleavage of eIF4GI but correlated with the cleavage ability. On the other hand, the data indicated that the impaired growth of FMDV mutants was associated with its ability to shut off host cell cap-dependent protein synthesis.

### Expression of type I and type III IFN induced by mutant FMDVs in PK-15 cells

**Fig. 4:**
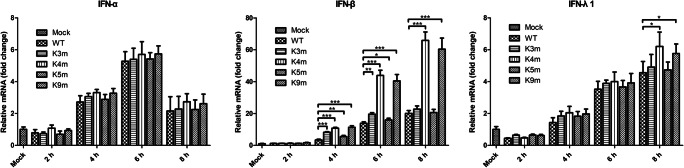
Type I and type III IFN expression induced by the WT and FMDV mutants. PK-15 cells were infected with WT and mutant FMDVs at 1 MOI for 0, 2, 4, 6, and 8 h. Total cellular RNA was prepared, and RT-qPCR was performed to examine the mRNA levels of IFN-α, IFN-β, and IFN-λ1. Results were obtained from three independent experiments

As mentioned previously, a weakened FMDV with deletion of L gene showed an inability to suppress the expression of IFN-α/β mRNA and block host innate immune defenses (de Los Santos et al. 2006, 2007; Wang et al. 2011a). To study whether the attenuated phenotype was related to the high induction of type I and type III IFN, the mRNA levels of IFNs induced by mutant FMDVs were measured in PK-15 cells by RT-qPCR. As shown in Fig. 4, FMDV infection caused the expression of IFN-α, IFN-β, and IFN-λ1, with the highest level of IFN-β, revealing that IFN-β may be a dominant IFN to exert anti-FMDV activity in PK-15 cells. The four mutant FMDVs induced basically similar mRNA levels of IFN-α or IFN-λ1 than the parental virus. In sharp contrast, IFN-β mRNA productions resulted by K4m or K9m were found to be significantly upregulated compared with that seen with WT infection. K3m or K5m triggered higher IFN-β expression compared with the WT virus but stimulated significantly lower IFN-β expression than K4m or K9m. These results suggested that the reduced ability to suppress IFN induction in host cells was another factor to be responsible for the delayed replication of Lb AUG-modified FMDVs in IFN-competent cells.

### Influence of Lb AUG modifications on L protein synthesis in vivo

**Fig. 5:**
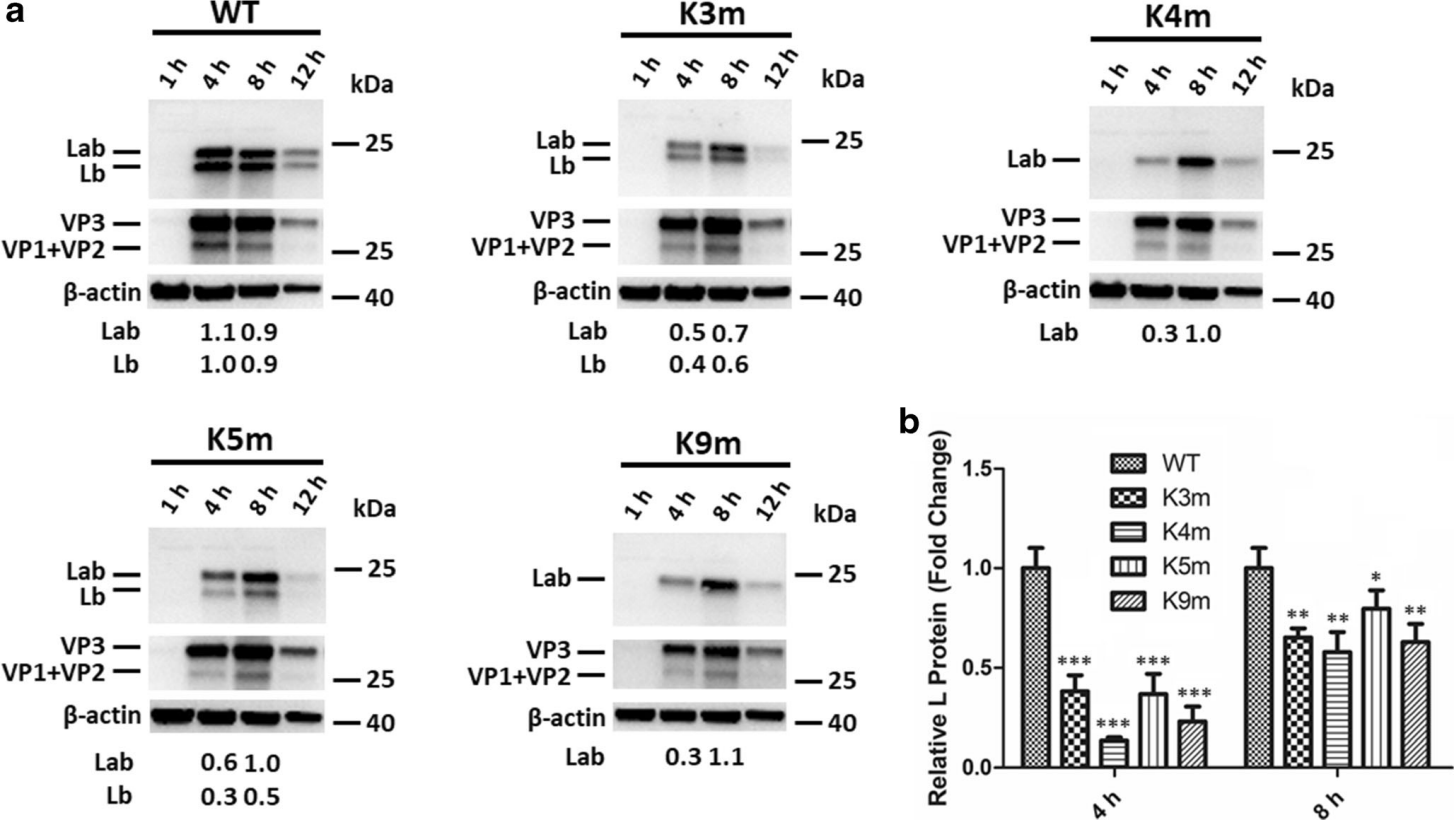
Synthesis of L protein during infection of WT and mutant FMDVs (**a** and **b**). **a** BHK-21 cells were infected with WT and mutant FMDVs (MOI = 1) for 1, 4, 8, 12, and 20 h. The expression level of L and viral structural proteins were determined by Western blotting using anti-L and anti-FMDV antibodies. **b** The total yield of the two forms of L proteins was analyzed. The band intensities were determined by densitometry and quantitated relative to the yield of Lb protein in WT-infected cells, which was set at 1.0

Among the proteins encoded by FMDV, L protein has been proved to be involved in eIF4GI cleavage and suppression of IFN expression. Therefore, we hypothesized that the different eIF4GI cleavage efficiencies and distinct abilities to suppress IFN expression between the WT and mutant FMDVs were correlated with the amount of L protein synthesis. To demonstrate this hypothesis, L protein expression was examined during FMDV infection. Unexpectedly, the anti-L serum prepared in our lab could not identify the L protein synthesized in PK-15 cells. So, we performed this assay in BHK-21 cells (Fig. 5a). For the parental virus O/HN/CHN/93, no obvious difference was detected in the expression between Lb and Lab protein, indicating that the initiation frequency for protein synthesis at the two AUG sites was approximately equal. As expected, only one form of L protein, Lab protein, was expressed for K4m and K9m. Most surprisingly, both forms of L proteins were detected for K3m and K5m, the same to that during WT FMDV infection, suggesting that non-AUG codons UGG and CUG could be used to initiate Lb synthesis in the current sequence environment, but the initiation frequency was obviously impaired. In addition, we also observed that mutating Lb AUG to UGG did not alter the ratio of the two products while mutating Lb AUG to CUG increased Lab product. The results above demonstrated that Lb AUG mutations altered the usage of the two initiation sites. Subsequently, the product yield of the two forms of L proteins was then summed to calculate the total expressed L proteins at different time points during the infection cycle. The outcome showed that in the early infectious cycle (4 hpi), Lb AUG modifications markedly reduced the total yield of all products initiated at the Lab and Lb sites. Especially, the L protein synthesis during K4m or K9m infection decreased to 15% of that in WT-infected cells, indicating an extremely low protein translation efficiency for K4m or K9m. Subsequently, an increase in the expression of overall L proteins was observed in the late infectious cycle (8 hpi), as exemplified by Fig. 5b. These data confirmed that modifications of Lb AUG interfered with the synthesis of viral L proteins through changing the selection of the alternative initiation sites in L sequence, resulting in reduced abilities of FMDV mutants to antagonize the host antiviral response.

### Effect of Lb AUG modifications on in vitro translation activity

**Fig. 6:**
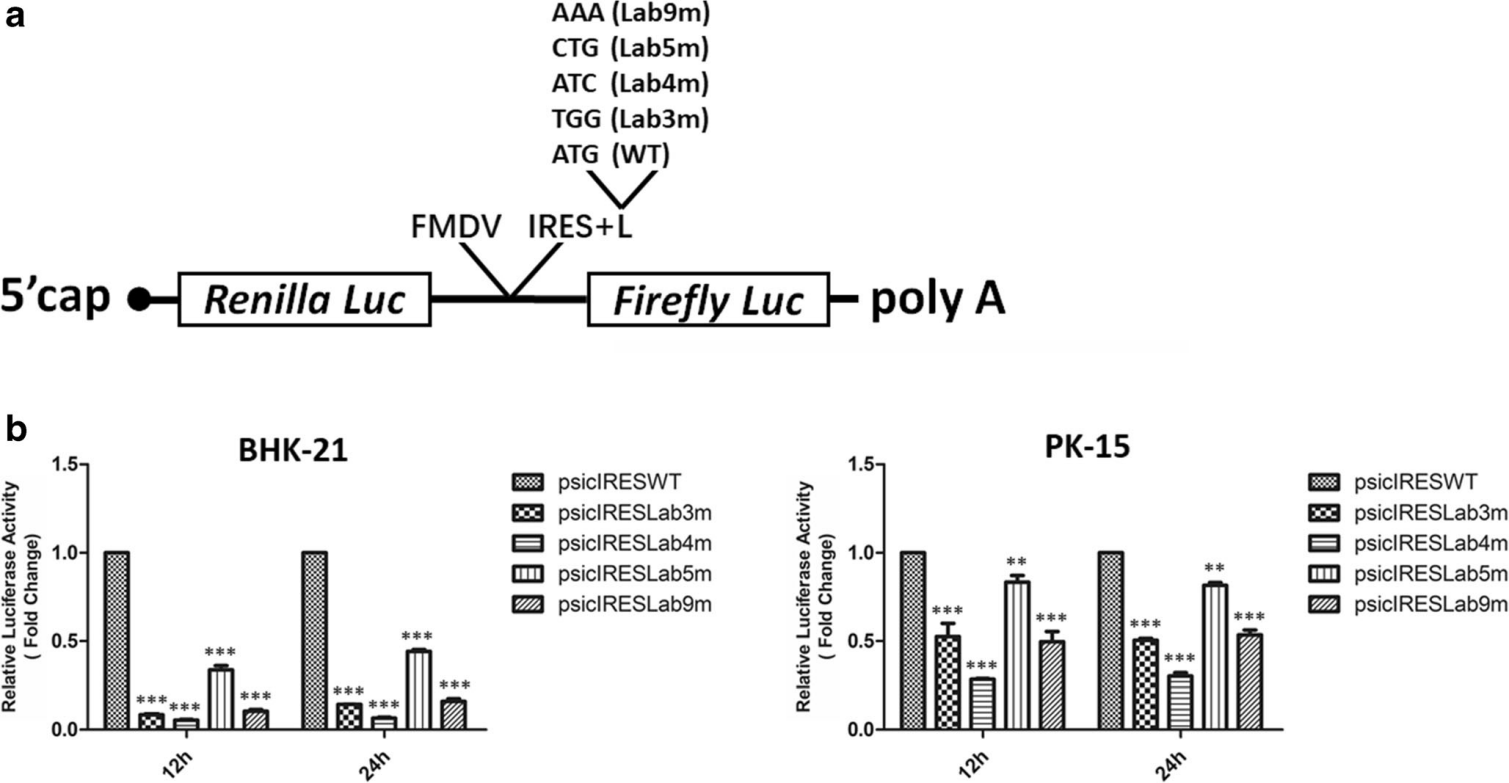
Influence of Lb AUG modifications on translation activity in vitro (**a** and **b**). **a** Schematic illustration of dual-luciferase reporter plasmids. The IRES sequence upstream of the *firefly* luciferase coding sequence in plasmid psiCHECK was fully replaced by the corresponding IRES and L regions of parental and mutant FMDVs, producing five newly constructed reporter plasmids. **b** BHK-21 or PK-15 cells were transfected with constructed reporter plasmids (0.5 μg). The dual-luciferase reporter activities were detected after transfection for 12 and 24 h. This assay was performed at least three times

The above experiments indicated that Lb AUG modifications reduced viral protein translation efficiency. In order to further confirm this result, the effect of Lb AUG mutations on in vitro protein translation was evaluated. We constructed dual-luciferase reporter plasmids in which the IRES sequence in plasmid psiCHECK was completely replaced by the corresponding IRES and L regions of FMDV mutants (Fig. 6a). BHK-21 or PK-15 cells were respectively transfected with the constructed luciferase reporter plasmids (psicIRESWT, psicIRESLab3m, psicIRESLab4m, psicIRESLab5m, and psicIRESLab9m), and the luciferase activities were detected. It was observed that each modified Lb AUG led to a decreased yield of luciferase activities in both BHK-21 and PK-15 cells (Fig. 6b). We found that the decreased translation efficiency in BHK-21 was different from that in PK-15 cells but nearly in parallel with it, possibly due to the distinct translation system in these two cell lines. These results demonstrated that Lb AUG modifications indeed had a negative influence on protein translation activity.

## Discussion

Two alternative translation initiation sites are invariably noted in L coding sequence, and they are found to be responsible for the initiation of viral protein synthesis. Previous studies showed that modification of Lab AUG codon alone or the Lab AUG modification in combination with the lack of Lb region did not block virus viability (Belsham 2013; Cao et al. 1995). We also demonstrated that for FMDV O/HN/CHN/93 used in the present study, the individual mutation of Lab AUG was tolerated (date was not shown). Thus, these observations suggested that the presence of Lab AUG was not essential for virus replication. The modification of Lb AUG to UUU has been reported to abolish the replication of the O1K mutant (Cao et al. 1995). This may be related to that the Lb AUG is a more favorable site for translation initiation than the Lab AUG, and its modification might prevent virus viability. Inversely, in the present study, we found that mutating Lb AUG to a series of non-AUG codons did not affect virus viability, possibly due to the fact that the initiation frequency at the two AUG sites was approximately equal for FMDV O/HN/CHN/93, and the initiation at Lab AUG could proceed when Lb AUG was eliminated, as shown by Fig. 5a. To sum up, these results indicated that neither Lab AUG nor Lb AUG was essential for FMDV viability.

Though Lb AUG modifications did not prevent viral viability, the growth characteristics of modified FMDVs were influenced. We found that these mutant FMDVs had a relatively minor difference in plaque morphology and replication ability in BHK-21 cells compared with the WT virus, whereas the growth of virus K4m or K9m was significantly impaired in PK-15 cells, indicating that Lb AUG modifications restricted FMDV proliferation in a cell-specific manner. Since it had been proved that BHK-21 was an IFN-incompetent cell line while PK-15 was an IFN-competent cell line (de Los Santos et al. 2006), the restriction phenotype of FMDV mutants in PK-15 cells was possibly caused by their weakened ability to inhibit IFN expression. An earlier study reported that the ability to form plaques of FMDV A12-LLV2 which markedly reduced growth rate in cells with a competent IFN system was associated with its inability to suppress the expression of IFN-α/β and abolished eIF4GI processing (Chinsangaram et al. 1999). These important findings prompted us to detect the integrity of eIF4GI and IFN transcripts in PK-15 cells infected with Lb AUG-mutant FMDVs. Our results presented here showed that a delayed eIF4GI cleavage and increased induction of type I and type III IFN expression were clearly confirmed for virus K4m or K9m, which was quite harmful to block host antiviral activity. Thus, we demonstrated that the restricted growth phenotype of the modified FMDVs in PK-15 cells correlated with the greatly reduced ability to inhibit host cell protein synthesis and block IFN induction.

Next, we explored the underlying causes for the attenuation of FMDV mutants. Given the crucial roles of L protein in eIF4GI cleavage and antagonizing host immune response (Gradi et al. 2004; Liu et al. 2015), we hypothesized that the total amount of L proteins was obviously influenced at least in K4m- or K9m-infected cells. Then, the effect of Lb AUG modification on L protein synthesis was analyzed. We observed that the product yield of total L proteins in K3m- or K5m-infected cells was markedly reduced by ~ 57% at 4 hpi and significantly increased to 72–83% at 8 hpi when comparing with that in WT-infected cells. In contrast, the amount of L proteins for K4m or K9m was reduced by ~ 85% at 4 hpi and increased to 56–61% at 8 hpi. Though all modified FMDVs resulted in reduced yields of L proteins during viral infection, the K4m or K9m appeared to cause a reduction to a greater extent throughout the processing of virus infection, which was consistent with our hypothesis above. In addition, the synthesis of two forms of L proteins was observed for K3m or K5m. However, K3m basically did not change the ratio of Lab/Lb, and K5m reduced the expression of Lb protein with a compensatory increase in the yield of Lab protein. In the case of K4m or K9m, only a single Lab protein was found, suggesting that the initiation at the Lb site was fully abrogated. These results were strong indications that not only the total expression of L proteins was severely influenced for the mutant FMDVs but also the individual yield of L protein was seen to be affected too.

As L protein expression was proportional to the utilization frequency of translation initiation sites, we could conclude that Lb AUG modifications influenced the relative selection of Lab and Lb initiation sites and thus regulated viral translation initiation efficiency, finally determining L protein synthesis and the outcomes of FMDV infection. The further examination of the influence of Lb AUG mutations on protein translation activity was performed in vitro using a dual-luciferase reporter assay. As was confirmed by the yield of luciferase activity in BHK-21 or PK-15 cells, all Lb AUG substitutions caused a delay in luciferase synthesis, suggesting that the in vitro protein translation was also reduced. Collectively, the in vivo and in vitro experiments proved that Lb AUG modifications changed protein translation efficiency, which were the decisive mechanism for viral attenuating phonotypes.

L protein remains the most thoroughly investigated determinant of FMDV pathogenesis. Over the last 20 years, several alternative approaches to modify L coding sequence have been examined for obtaining attenuated FMDV as follows: (1) A genetically engineered FMDV lacking L coding gene (A12-LLV2) is highly attenuated in both cattle and swine (Brown et al. 1996; Mason et al. 1997). (2) An FMDV mutant containing amino acid substitutions in a conserved domain of the L coding region (A12-SAP) is attenuated not only in vitro but also in vivo (de los Santos et al. 2009; Segundo et al. 2012). (3) Insertion mutagenesis studies have indicated that certain in-frame disruptions of L gene are sufficient to generate a FMDV with attenuated phenotype in cattle. However, none of these vaccine platforms is applied to the actual production of FMD vaccines. In the present study, we obtained four FMDV mutants in which the Lb initiation AUG was individually modified to a non-AUG codon. These mutant FMDVs grew almost as well as the WT virus in IFN-incompetent BHK-21 cells, a typical cell line used for FMD vaccine production, but two of them displayed impaired replication in IFN-competent PK-15 cells, suggesting that we successfully generated genetically engineered FMDVs with attenuated phenotype. In the case of virus escape, such FMDVs can be expected to proliferate poorly outside BHK-21 cells. The approach of precise engineering of FMDV with the modification of the initiation codon provides a safe platform to produce inactivated antigen vaccines.
